# Medical Opinion and Movement

**Published:** 1910-06-25

**Authors:** 


### Medical Opinion and Movement.

### Ileosigmoidostomy for Constipation.

The operation of ileosigmoidostomy, or short-circuiting the large intestine by implanting the ileum into the sigmoid, is already well known in this country. It was originally conceived by Mr. Arbuthnot Lane, and so far he has been the chief exponent of this method of treatment for cases of inveterate constipation, in which, as he himself puts it, the lower bowel has become a stagnant cesspool giving rise to continual auto-intoxication. This form of heroic treatment has been regarded somewhat askance by the rest of the profession, and it is therefore interesting to note that a French surgeon, M. Morestin, has carried out the operation successfully on a patient suffering from severe and chronic constipation which failed to be relieved by ordinary methods of treatment. He reported the case at a recent meeting of the Société de Chirurgie. There was marked ptosis of the large intestine, with considerable dilatation, the walls being very thin. Since the operation the patient has one or two evacuations of the bowels daily, and the general condition of health is stated to be excellent.

### Albuminous Milk for Infants.

At a recent meeting of the Berlin Society of Medicine Drs. Finkelstein and Meyer brought forward their new method of feeding infants affected with dyspeptic troubles and unable to digest the ordinary milk food. On the ground that such troubles are due to intestinal fermentations, they have endeavoured to discover what is the constituent of milk which determines fermentation, and they conclude that the carbohydrate lactose is the chief cause. In cases of fermentation the stools give an acid reaction, whereas an increase of casein in the diet tends to an alkaline reaction. There is often a large proportion of undigested fat in the stools of dyspeptic infants, but this disappears if the food consists of casein and cream to the exclusion of lactose, so that the presence of lactose appears responsible for the intolerance of fat. Arguing on these lines, therefore, they have designed a food consisting almost entirely of the casein and fat of milk, which they prepare in the following manner: A litre of milk is coagulated or converted into junket by means of rennet ferment, and the coagulated casein, containing also the fat of the milk, is broken up into half a litre of water and half a litre of butter-milk added to it. This so-called "albuminous milk" contains only about 14 grammes of sugar instead of 72, about the same amount of fat, and 27 instead of 12 grammes of casein. This milk preparation is given until the digestion is again in order. It is sufficient to maintain weight, but the deficiency in carbohydrate prevents any recovery in this respect. In the course of treatment they advise, therefore, the addition of some carbohydrate other than lactose, such as maltose or a combination of maltose and dextrine. During the last two years they have treated 150 infants by this method, and they claim to have obtained most successful results. At a subsequent meeting of the same society Dr. Langstein gave details of the excellent results he had obtained by this method, and several other members spoke in the same strain in the course of the discussion.

### Injections of Sulphurous Acid.

Various sulphur preparations have been employed in the treatment of pulmonary phthisis, and among them sulphurous acid in the form of inhalations on account of its bactericidal properties and its power of diffusion. Dr. E. Talini, of Milan, has conceived the idea of administering this substance by intramuscular injections. He first used 2 to 5 cubic centimetres of a normal solution of sodium chloride containing 0.5 per cent. of sodium bisulphite, and then gradually increased the strength to 5 per cent. He then attempted to employ a more efficacious solution by adding 1 per cent. sulphurous anhydride, and finally, in order to obtain a tonic action upon the heart, he added 5 per cent. of calcium chloride, so that eventually the solution for injection consisted of normal saline containing 5 per cent. sodium bisulphite, 1 per cent. sulphurous anhydride, and 5 per cent. calcium chloride. Five cubic centimetres were injected for adults and 3 cubic centimetres for children. The injections were made daily into the muscles of the buttocks. According to this author the treatment was attended by a general improvement in the patient's condition, an increase in body weight, a diminution in catarrhal symptoms, disappearance of night sweats, and a progressive diminution and final disappearance of the fever, even in advanced cases. When the condition is sufficiently improved the injections are given every other day, and later only twice a week. Even after 100 injections examination of the urine failed to show any trace of albumen. Unfortunately it is not stated whether the usual methods of treatment by diet, fresh air, etc., were adopted at the same time.

### Wassermann's Reaction for Syphilis.

An interesting discussion took place at a recent meeting of the Surgical Society of Berlin on the value of the Wassermann reaction for syphilis. Freudenberg pointed out that, without wishing to depreciate the assistance that the reaction may afford in clinical diagnosis, the greatest prudence must be used in drawing conclusions from the results reported from the different institutions and laboratories. He proceeded to illustrate the uncertainty of the data by recounting a number of cases in which he had sent blood-specimens to three or four different laboratories and had obtained entirely contradictory reports. In some cases he obtained contradictory results with specimens from the same patient, and sent to the same laboratory. Thus in a case of ulceration of the prepuce the first specimen was reported to give a positive reaction. Three days later a specimen sent to another institute was said to show a negative reaction. Five days later again specimens were sent to four different laboratories and three different reports were obtained, and the laboratory that had the first specimen and reported a positive reaction now called it doubtful! Dr. Müksam in discussing the question considered that differences are to be accounted for largely by the different methods of preparing the "extracts," and it was urged that a definite agreement should be arrived at as to the best method of carrying out the reaction. According to Dr. L. Michaelis, alcoholic extracts of the heart are preferable to all others, because with these doubtful reactions become negative, and it is certainly better to incline towards the negative than obtain a false positive reaction.

### Reduction of Shoulder Dislocations.

Gallois, in the *Bulletin Médical*, advises that the fourth step in the reduction of shoulder dislocations by Kocher's method should be omitted. After flexing the forearm to a right angle, with the elbow pressed against the side of the body, the hand is carried outwards, causing external rotation of the humeral head. The third step consists in maintaining the hand in this position while the elbow is moved forwards, inwards, and upwards. Kocher then advises that the hand should be carried rapidly towards the sound shoulder, and it is this last manœuvre that Gallois finds unnecessary. He argues that if proper gentleness and deliberation be used, the head of the humerus will return to its normal position in the glenoid cavity while the third movement is being carried out. If it be performed slowly and brought to a stop as soon as the greatest resistance is met with, after a few seconds' delay a click will be felt, which denotes the re-entry of the head of the humerus into the glenoid cavity. The essential point in the procedure is the avoidance of force and clumsy manipulation which will cause pain and so contract the muscles governing the joint. The patient should also not be told that an attempt is being made to reduce the dislocation, or he will certainly resist voluntarily. The author remarks that in jiu-jitsu dislocation of the opponent's shoulder is brought about by movements which are the exact reverse of those employed in the first three steps of Kocher's method. The fourth step of this method has, however, no place in the procedure, which fact again shows that it is unessential.

### Detection of Arsenical Poisoning.

In connection with a poisoning affair in France, in which Fowler's solution was the cause of death, the toxicological examination of the body was made 19 months after poisoning, and notwithstanding that long period of interment, most of the organs, especially the muscles, were found to be in a remarkably good state of preservation. In tabulating the results according to the decreasing content of arsenic, the following quantities, in milligrams, of arsenic per kilogram of the organ were found: Liver, 360; intestines, 82; stomach, 42; kidneys (mean), 28; nails, 23; lungs, 26; hair, 15; heart, 14; muscles, 11; bone (scapula), 5; brain, 2. The brain is always the organ which stores least of the poison. Regarding the quantities of fat found, the following percentage figures are given by the author: Kidneys (largest) 68, (smallest) 53; stomach, 64; intestines, 62; heart, 41; muscles, 29; liver, 28; scapula, 22. The fatty degeneration of the various parts examined is manifest. It is extremely marked in the kidneys; very evident also in the liver, which, according to Gorup Besanez and other authorities, does not contain more than 3 per cent. of fat in the normal state.

### Bromidrosis.

The distressing and repulsive condition in which a profuse and very foul-smelling sweat is excreted by the skin of the feet is one which is not always easy to cure. Major Hale, of the Royal Army Medical Corps, contributes to the *Journal* of that body an account of the plan he has found most useful. He regards hyperidrosis and bromidrosis as different stages of one condition; the former, if neglected, leads to the latter. He inclines to the belief that a specific organism may be at work, and his treatment is largely a system of careful antisepsis. He advises that all socks should be soaked for an hour in perchloride solution (1 in 2000), well rinsed in hot water three times, and then thoroughly washed. The insides of the soles, vamps, quarters, and counters of boots are to be painted with a solution of 1 part of salicylic acid in 4 parts of methylated spirit, applied with a camel-hair brush. The feet are also well washed, then dried, and painted with the same solution, which must reach every crevice and cranny between the toes. A clean, dry pair of socks is then donned, and the feet are repainted the next day. This usually suffices to dispel bromidrosis at once, and relieves the patients also. The author agrees with other authorities that the flat-footed are more subject to this condition, which is undoubtedly brought on by neglect of cleanliness and wearing dirty socks which have been dried frequently without being washed.

### Pneumonia at Birth.

A very curious case of antenatal lobar pneumonia is reported in the *Journal of Obstetrics and Gynæcology of the British Empire* by Dr. Alexander Don. The infant was the fourth child of healthy parents; the mother had not been in contact with any case of pneumonia, nor had she suffered from any form of illness except that during the last four days of pregnancy she had been cold and shivery and for the same space had felt no fœtal movements. After a rather short labour the baby was born still enveloped in the membranes, which were cut open with scissors by the attending physician. As it showed little respiratory activity it was rubbed and slapped in the usual way, and at once coughed up some brownish mucous material. This the author is confident could not have come from the swallowing of any maternal discharges. About two hours after the birth the nurse noticed that the child was cyanosed and breathing with difficulty, and when the doctor reached it he found it whining with every respiration. Moist crackling râles and pleural friction were detected on auscultation in the lower half of the left chest; the pericardium was thought not to be involved. Some more brownish expectoration was coughed up. About twenty hours after birth the infant died, and a swab was immediately taken from the child's mouth. This was found to contain pneumococci in large numbers, with a few staphylococci. Antenatal pneumonia is not so very rare when the mother also is infected with the disease, but even then septic forms are the most usual. A pneumococcal inflammation such as this with a healthy mother must be almost unknown.

### Mercurial Injections and the Teeth.

In *Le Progrès Médical* Milan describes a condition of the teeth which results from the application of mercurial injections to patients suffering from syphilis. While under treatment the patient notices that there is a sensation of pain in the teeth on breathing cold air or on drinking cold or hot fluids. This sensation increases with the prolongation of treatment, but goes away when the mercury is stopped. On examination the teeth are seen to be grooved from side to side, the ivory being absent in the grooves and the sensitive dentine exposed along the gingival margins. The bottom of the groove is seen to be white, shining, hard and polished, triangular in shape, and showing no resemblance to true caries. These manifestations are more commonly met with in the front teeth, and occur for the most part on the labial surfaces. Patients vary in age from 20 to 50, and are otherwise of healthy appearance. While many attempts have been made to explain the lesions, such as chemical action and too severe brushing of the teeth, the author describes a case in which the evidence is conclusive that the condition occurs while the patient is under mercurial influence, and ceases as soon as that influence is removed. He believes that it may occur in persons whose habits are quite correct from the hygienic standpoint, in which case it takes the place of the ordinary stomatitis of those whose methods are not all that is desirable. Mercury should therefore be carefully administered, the drug being stopped as soon as the teeth show signs of becoming tender. At the same time these last should be kept as clean as possible by frequent brushing and by attention to the ordinary dental hygienic details, which are of paramount importance in patients undergoing treatment with mercury in any form.

### Neoplastic Stenosis of the Colon.

As reported in *La Riforma Medica*, Minerbe and Giocomo were able to demonstrate that the colon was obstructed in a case of lympho-sarcoma of that organ and the retroperitoneal glands by inflating the intestine with a bicycle pump attached to a rectal tube. The piston-stroke imparted such a strong impulse to the air in the colon that an expansive pulsation was transmitted to the tumour, which could be felt with the hand on the abdomen. This pulsation the authors consider a pathognomonic sign of obstruction of the descending colon. They have carried out a series of observations both on living and dead bodies, and now maintain that the following conditions must be present for the test to be carried out properly. In the first place the sphincter ani must be intact, and, secondly, some degree of stenosis of the intestine must be present as the result of degeneration. There must, thirdly, be some degree of induration of the colon walls following infiltration, and, fourthly, only a portion of the circumference of the bowel must be invaded or the test will fail owing to the inextensibility of the intestine. Patients with abnormally thick abdominal walls, or in whom the colon is deeply situated, or whose abdominal walls are much contracted, are all unfavourable subjects to whom to apply the test. A bowel distended with fæces also prevents a successful result to the application of the test. The authors likewise mention that by a similar method applied to the stomach with an œsophageal tube that organ can be made to stand out so that its boundaries can be recognised through the walls of the abdomen.

### Ping-Pong Ball Fracture.

A very curious and hitherto undescribed lesion of the infantile skull has come under the observation of Dr. Luckett, who reports it in the *Annals of Surgery*. The patient was five and a half months old, and had fallen whilst asleep off a low couch on to the floor, a distance of less than two feet. The child was not unconscious, but was irritable to a very marked degree and would not take the breast. It had vomited profusely immediately after the accident. The eyes were normal, as also the temperature and pulse. There was no muscular rigidity nor any local manifestations of intracranial pressure. Over the left parietal bone a three-cornered depression was discernible, both to sight and touch; there was no injury to the overlying soft parts. On the operating table an incision was made over the depressed area, and a three-sided pyramidal depression was found just like that produced on a thin celluloid ping-pong ball. In the centre of this a very small trephine opening was made and a hook passed under the edge of the bone, which was one-sixteenth of an inch in thickness. On making traction the depression resolved with a sudden pop just as by manipulation an indented ping-pong ball will do. Careful exploration with a flat silver probe between the dura mater and the skull revealed no fracture anywhere. Recovery was uneventful. The case seems to have been undoubtedly one of cerebral imitation from indentation of the very thin flexible skull without fracture. A somewhat similar case is reported in a recent number of the *British Medical Journal* by Dr. Cossham, but in this case the depression was caused during labour; there were no symptoms, and operation was performed successfully ten days after birth.

